# Suppression of prostate cancer progression by cancer cell stemness inhibitor napabucasin

**DOI:** 10.1002/cam4.675

**Published:** 2016-02-21

**Authors:** Yiming Zhang, Zhong Jin, Huimin Zhou, Xueting Ou, Yawen Xu, Hulin Li, Chunxiao Liu, Bingkun Li

**Affiliations:** ^1^ Department of UrologyZhujiang HospitalSouthern Medical UniversityGuangzhouChina; ^2^ Department of GastroenterologyThe First Affiliated Hospital of Clinical Medicine of Guangdong Pharmaceutical UniversityGuangdong Pharmaceutical UniversityGuangzhouChina; ^3^ Department of RespiratoryThe Third Affiliated Hospital of GuangZhou Medical UniversityGuangZhou Medical UniversityGuangzhouChina

**Keywords:** Cancer cell stemness inhibitor, chemotherapy, napabucasin, prostate cancer, stemness‐high cancer cells

## Abstract

A small population of cells with stem cell‐like properties in prostate cancer (PCa), called prostate cancer stem cells (PrCSCs) or prostate stemness‐high cancer cells, displays highly tumorigenic and metastatic features and may be responsible for the therapy resistance. A small molecule, napabucasin (BBI608), recently have been identified with suppression of stemness‐high cancer cells in a variety of cancers. However, the effects of napabucasin on PCa cells as well as PrCSCs isolated from PCa cells have not yet been defined. The effect of napabucasin on PCa cells in cell proliferation, colony formation, and cell migration in vitro were measured by MTS, colony formation assay, and Transwell, respectively. Flow cytometry was employed to evaluate cell cycle and cell apoptosis, and the effect on tumorigenesis in vivo was examined by tumor growth assays. Furthermore, the role of napabucasin on self‐renewal and survival of PrCSCs was evaluated by their ability to grow spheres and cell viability assay, respectively. Western Blot and qRT‐PCR were used to determine the effect of napabucasin on the expressions of stemness markers. Decrease in cell viability, colony formation, migration, and survival with cell cycle arrest, higher sensitivity to docetaxel in vitro, and repressed tumorigenesis in vivo was observed upon napabucasin treatment. More importantly, napabucasin can obviously inhibit spherogenesis and even kill PrCSCs in vitro. Downregulation of stemness markers was observed after PrCSCs were treated with napabucasin. This study demonstrates that napabucasin may be a novel approach in the treatment of advanced PCa, specifically for castration‐resistant prostate cancer (CRPC).

## Introduction

Prostate cancer (PCa) is the second leading cause of cancer death in men worldwide 1. However, the prognosis for patients at advanced stage is still poor. Although chemotherapy or androgen deprivation therapy can induce partial or almost complete cancer regression temporarily in patients suffered from advanced disease, recurrent PCa is almost inevitable and becomes resistant to further therapies.

Currently, more and more studies have proposed that PCa includes a small population of cells that display unlimited self‐renewal potential and tumor‐initiating capacities 2, 3, 4, 5. These cells are often termed as prostate cancer stem cells (PrCSCs), which can survive from chemotherapy or radiotherapy and are suggested to be responsible for the development of castration‐resistant disease and the poor prognosis of patients in advanced staged PCa 3, 6, 7. Therefore, PCa tumor‐initiating cells are regarded as a potential therapeutic target.

Napabucasin (BBI608) is a newly found small molecule with the ability to inhibit gene transcription of STAT3, which was able to suppress cancer stemness properties and induce cell death 8. Li et al 8. had reported that napabucasin inhibited the expressions of stemness markers and kill stemness‐high cancer cells isolated from several kinds of tumors except PCa. Hence, we intended to clarify the potential roles of napabucasin on PrCSCs as well as on nonstem cancer cells.

In our study, our results showed that napabucasin not only inhibited cell proliferation, cell motility, cell survival, colony formation ability, and tumorigenic potential of PCa cells, and increased cell apoptosis and sensitivity to docetaxel, but also effectively block sphere formation of PrCSCs and kill them in vitro and in vivo as well as inhibit stemness gene expression. Taken together, napabucasin may be a novel approach to suppress cancer progression and improve prognosis for advanced PCa.

## Materials and Methods

### Cell lines and cell culture

The PCa cell lines (22RV1and PC‐3) were supplied by the Cell Bank of the Chinese Academy of Sciences (Shanghai, China) that had been authenticated by STR profiling (See additional supporting information). Cells were maintained in RPMI‐1640 supplemented with 10% fetal bovine serum (FBS, Gibco, Carlsbad, California, USA), penicillin (100 Units/mL) and streptomycin (100 mg/mL) (Life Technologies, Carlsbad, California, USA). All of the cells were grown in a humidified incubator at 37°C with 5% CO_2_.

### Drugs

The cancer cell stemness inhibitor napabucasin was purchased from MedChem Express (New Jersey, USA) while the docetaxel was obtained from Meilun (Dalian, China).

### Sphere culture

Suspension cultures to generate spheroids were performed as described in Dubrovska et al 9. Briefly, single cells were plated at 1000 cells/mL on 10‐cm low attachment dishes with an ultralow attachment surface (Corning, New York, USA). Cells were grown in RPMI‐1640 supplemented with 4 *μ*g/mL insulin (Sigma, KGaA, Darmstadt, Germany), B27, 20 ng/mL \epidermal growth factor (EGF), and 20 ng/mL basic fibroblast growth factor (FGF) (Invitrogen, Carlsbad, California, USA). Spheres were collected after 7–14 days.

For sphere formation assay**,** cells were disassociated, counted, and transferred to ultra low attachment plates (Corning) in suspension culture medium as described above. Sphere growth was scored by counting the number of spheres possessing >50 cells.

### Cell proliferation assay and colony formation assay

For cell proliferation assay, cells were seeded in 96‐well plates at 2.0 × 10^3^cells/well in a final volume of 100 *μ*L and incubated overnight. The viability of cells was determined with CellTiter 96 non‐radioactive cell proliferation assay (MTS) (Promega BioSciences, Madison, Wisconsin, USA) following the manufacturer's protocol. For colony formation assay, cells were placed in a six‐well plate and maintained in RPMI‐1640 supplemented with 10% FBS for 2 weeks. The colonies were fixed with 4% paraformaldehyde, stained with 0.1% crystal violet and counted.

### Cell apoptosis assay by flow cytometry

For cell apoptosis assay, cells were disassociated and plated in 6‐well plates at 2 × 10^5^ cells/well. After incubated at 37°C for 48 h, the cells were collected, washed with PBS, and then analyzed with Annexin V‐FITC and PI (Keygen, Jiangsu, China) staining in a FACSCaliber BD flow cytometery.

### Cell migration assay

Migration assay was performed by suspending cells in serum‐free RMPI‐1640 medium and seeded them into the upper chambers of Transwell (Corning). The lower chamber of each well was added with 500 *μ*L RPMI1640 with 40% FBS. After incubated at 37°C for 18 h, cells were fixed and stained with the nonmigratory cells on the upper chamber were removed. Stained cells were visualized by light microscopy and counted in 10 random high‐power fields.

### Cell cycle analysis

For analyzing the cell cycle, cells were fixed with 70% ethanol in PBS at 4°C overnight, and then treated with ribonuclease to digest RNA and stained with 50 *μ*g/mL of PI. The cell cycle was analyzed by FACSCaliber BD flow cytometry.

### Chemoresistance analysis

For chemosensitivity assay, cells were treated with a series of different concentrations of docetaxel (Meilun, Dalian, China) (0, 2.5, 5, 10, 25, 50, and 100 nmol/L) for 48 h. The cell viability after docetaxel exposure was measured using the same method as MTS and the half inhibition concentration (IC_50_) of docetaxel was also calculated.

### Isolation of side population (SP) cells

The 22RV1 cells were harvested in RPMI‐1640 containing 2% FBS. Cells were added with 5 *μ*g/mL Hoechst33342 (Life Technologies) in the presence or absence of 50 *μ*mol/L verapamil (Sigma), and then incubated at 37°C for 90 min. After incubation, the cells were washed with ice‐cold 1 × PBS three times. Prior to analysis, propidium iodide (2 *μ*g/mL) (Sigma) was added immediately to discriminate dead cells. SP cells were then separated by BD Influx cell sorter (BD, Franklin Lakes, New Jersey, USA).

### Isolation of CD133^+^/CD44^+^ cells

For sorting cells, cells were dissociated with Accutase (Stem Cell Technologies, Vancouver, Canada), washed twice with PBS and processed for CD44 (Miltenyi Biotec, Cologne, German) and CD133 (Miltenyi Biotec, Cologne, German) multicolor staining, along with appropriate negative controls and single‐color positive controls. The CD44^+^/CD133^+^ populations were sorted out by a BD FACS Diva cell sorter (BD, USA).

### Quantitative real‐time PCR (qRT‐PCR)

Total RNA was isolated with TRIzol reagent (Invitrogen) and the cDNAs were synthesis with the reverse‐transcription kit (Takara, Japan). The quantitative analysis was performed using the LightCycler^®^ 480 SYBR Green I Master (Roche, Basel, Switzerland) on a LightCycler^®^ 480 System (Roche) according to the manufacturer's instructions. The relative mRNA expression was calculated using the 2^−ΔΔCt^ comparative CT method normalized to GAPDH and control. Primers sequences used were seen in Table 1.

**Table 1:** List of the primer sequences for PCR studies

Target	Sequence(5′‐3′)
KLF4	Forward: ACGATCGTGGCCCCGGAAAAGGACC
	Reverse: TGATTGTAGTGCTTTCTGGCTGGGCTCC
*β*‐catenin	Forward: CATCTACACAGTTTGATGCTGCT
	Reverse: GCAGTTTTGTCAGTTCAGGGA
Survivin	Forward: AGGACCACCGCATCTCTACAT
	Reverse: AAGTCTGGCTCGTTCTCAGTG
C‐myc	Forward: GCGTCCTGGGAAGGGAGATCCGGAGC
	Reverse: TTGAGGGGCATCGTCGCGGGAGGCTG
NANOG	Forward: TGAACCTCAGCTACAAACAG
	Reverse: TGGTGGTAGGAAGAGTAAAG
GAPDH	Forward: TGGTGAAGACGCCAGTGGA
	Reverse: GCACCGTAAGGCTGAGAAC

### Western blot

A standard western blot protocol was used and the details can be seen in our previous studies 10. The primary antibodies used and dilutions were as follows: *β*‐catenin (1:1000, Proteintech, Chicago, USA), Klf‐4 (1:1000, CST, Boston, USA), Nanog (1:1000, CST), survivin (1:1000, CST), C‐myc (1:1000, CST), and GAPDH (1:1000, Proteintech). The secondary antibody was HRP‐conjugated goat anti‐rabbit IgG (1:5000, CST).

### In vivo therapeutic studies

A total of 1 × 10^6^ PC‐3 cells or 8 × 10^6^ 22RV1 cells in 100 *μ*L of PBS were injected subcutaneously into dorsal flanks of an immunodeficient nude mouse. The animals were treated i.p. with napabucasin(40 mg/kg), docetaxel (10 mg/kg), or PBS q3d once the tumors have reached 50 mm^3^. The tumor volume (TV) was calculated every 4 days according to the following standard formula: TV (mm^3^) = length × width^2^ × 0.5. All of the experiments on mice were approved by the local ethics committee of Southern Medical University.

To determine the effect napabucasin on stem‐like properties in vivo, animals were sacrificed and the tumors were removed in sterile condition. Tumors were disassociated into single‐cell suspensions and counted. Live cells were cultured in suspension culture media for determining the ability of spherogenesis. The media was changed every 3 days, and sphere growth was determined after 10–14 days in culture by counting the number of spheres possessing >50 cells.

### Statistical analysis

SPSS 20.0 software (SPSS Inc, Chicago, USA.) was used to perform the statistical analysis. All results were given as the mean ± standard deviation (SD) of three independent experiments, and the significance of the differences among three groups was calculated using one‐way analysis of variance (ANOVA) while Student's *t*‐test was used to ascertain the significance between two groups. A threshold of *P *< 0.05 was considered to be statistical significant.

## Results

### Napabucasin inhibited PCa cell proliferation, cell motility, cell survival, colony formation ability and induced cell apoptosis

**Figure 1:**
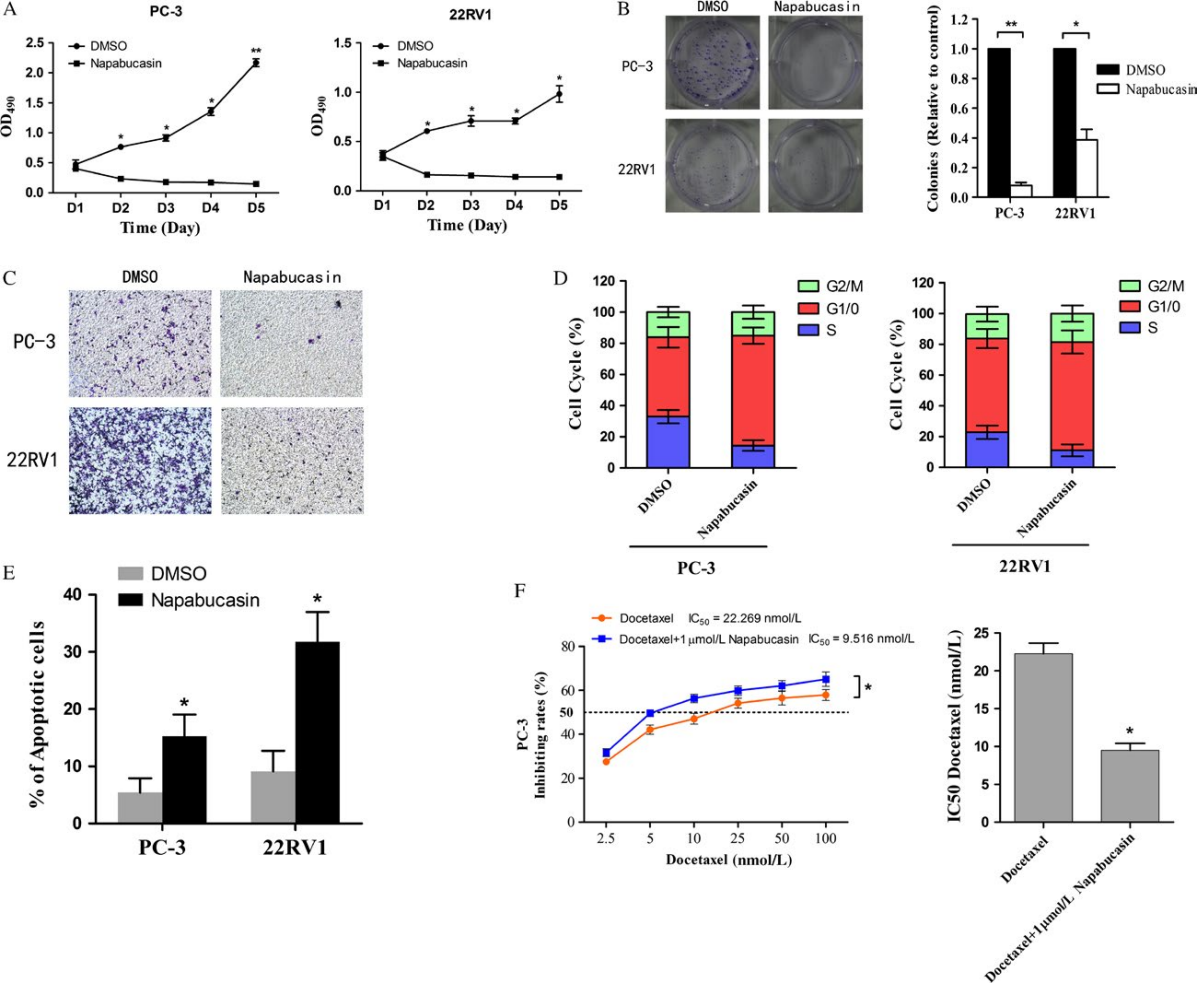
Napabucasin inhibited prostate cancer (PCa) progression effectively and increased the sensitivity to docetaxel in PCa. (A) The proliferation of PC‐3 and 22RV1 cells treated with 1 *μ*mol/L napabucasin were significantly decreased from day 2 to 5 compared with the control group (*P *< 0.05). The data shown are the means ± SD of a representative experiment performed in triplicate (*n* = 3). (B) Representative images of colony formation assay in PCa cell lines (PC‐3 and 22RV1) and histological analysis of the colony number showed that the colony formation of PCa cell lines were dramatically decreased after treated with 1 *μ*mol/L napabucasin for 24 h (*P < 0.05; **P < 0.01; *n* = 3). (C) The migration activity of the cells in the napabucasin group was reduced obviously compared with the control cells (*P *< 0.05). (D) The results of flow cytometry showed that PCa cells treated with napabucasin exhibited an increased rate of the G1‐phase and a decrease in the S‐phase (*P *< 0.05; *n* = 3). (E) In accordance with the inhibition of the cells survival by napabucasin, cells in the napabucasin group exhibited an increased proportion of apoptosis compared with the control group (*P *< 0.05; *n* = 3). (F) Growth curve of the cells in docetaxel at six different drug concentrations. Chemosensitivity assays showed that napabucasin can obviously increase the sensitivity of PC‐3 cells to docetaxel (*P *< 0.05; *n* = 3) with a lower IC_50_ values in napabucasin group than control group (*P *< 0.05; *n* = 3).

The antiproliferative activity of napabucasin against the PCa cell lines PC‐3 and 22RV1 was examined. As shown in Figure 1A, napabucasin inhibited cell proliferation in PC‐3 cells and 22RV1 cells at 48, 72, 96, and 120 h (*P *< 0.05). Cell motility and colony formation ability were closely correlated with the process of tumor metastasis. As shown in Figure 1B and C, napabucasin significantly decreased colony formation and cell motility ability of PCa cell lines in vitro (*P *< 0.05).

The cell cycle is a critical characteristic that could accurately reflect the cell survival and apoptosis of cancer cells. To examine whether napabucasin have an effect on the cell cycle of PCa cells, flow cytometry was used. As shown in Figure 1D, there was a significant decrease in the rate of S‐phase after treatment with napabucasin (*P *< 0.05). Moreover, our results showed an increased rate of apoptosis in PCa cells 24 h after napabucasin treatment (Fig.1E, *P* < 0.05).

### Napabucasin increased the sensitivity of PCa cells to Docetaxel

To determine whether napabucasin influenced the sensitivity of PC‐3 cells to docetaxel, these cells were treated with different concentrations of docetaxel for 48 h and detected by the MTS assay. As shown in Figure 1F, docetaxel resulted in more effective inhibition of proliferation in PC‐3 cells treated with 1 *μ*mol/L napabucasin than in those with Dimethyl sulfoxide (DMSO) (control) (*P *< 0.05). The IC50 for docetaxel in those cells treated with DMSO was 22.269, but was 9.516 in napabucasin‐treated cells (*P *< 0.05).

### Napabucasin inhibited PCa tumor growth in vivo

**Figure 2:**
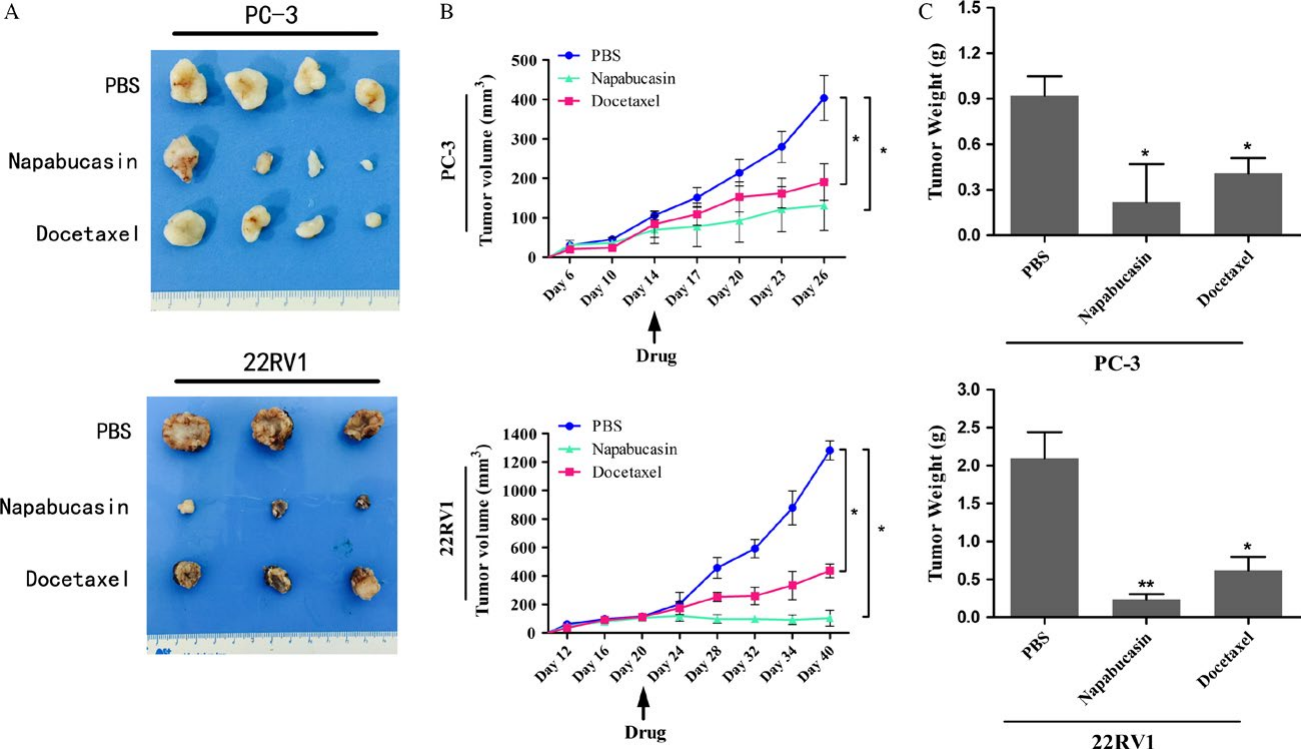
Napabucasin suppressed the tumor growth on prostate cancer (PCa) mouse xenograft models. Immunosuppressed mice with established human PCa (PC‐3 and 22RV1) were given napabucasin (40 mg/kg) by i.p., docetaxel (10 mg/kg), or PBS (control)q3d. (A) The mice treated with napabucasin or docetaxel showed marked reduction in tumor growth compared with the mice treated with PBS (control). (B) The volume of the tumors was significantly lower in mice treated with napabucasin or docetaxel than in the control group mice (*P *< 0.05). Moreover, a lower volume of tumors was showed in the 22RV1 xenograft models treated with napabucasin than those treated with docetaxel(*P *< 0.05). Each data point represents the mean ± (SD) of 3~4 mice. (C) The weight of the tumors was significantly decreased in napabucasin‐ or docetaxel‐treated mice than in PBS‐treated mice (control) (*P < 0.05; **P < 0.01).

To further validate the antitumor effects of napabucasin in vivo, PC‐3 cells or 22RV1 cells were firstly inoculated into nude mice. Once the tumors reached ∼50 mm^3^, the animals were treated i.p. with napabucasin at 40 mg/kg, docetaxel (10 mg/kg), or PBS (control) every 3 days. As shown in Figure 2A and B, napabucasin or docetaxel significantly reduced xenograft tumor growth and TV compared with PBS (*P *< 0.05). Notably, while no differences were observed between the napabucasin and the docetaxel groups in PC‐3 mouse xenograft models, the TV in napabucasin group was even lower than docetaxel group in 22RV1 mouse xenograft models (*P *< 0.05). Additionally, napabucasin or docetaxel also significantly reduced tumor weight compared with PBS (Fig.2C; *P *< 0.05).

### Napabucasin depleted stemness‐high cancer cells subpopulation of PCa cells in vitro and in vivo

**Figure 3:**
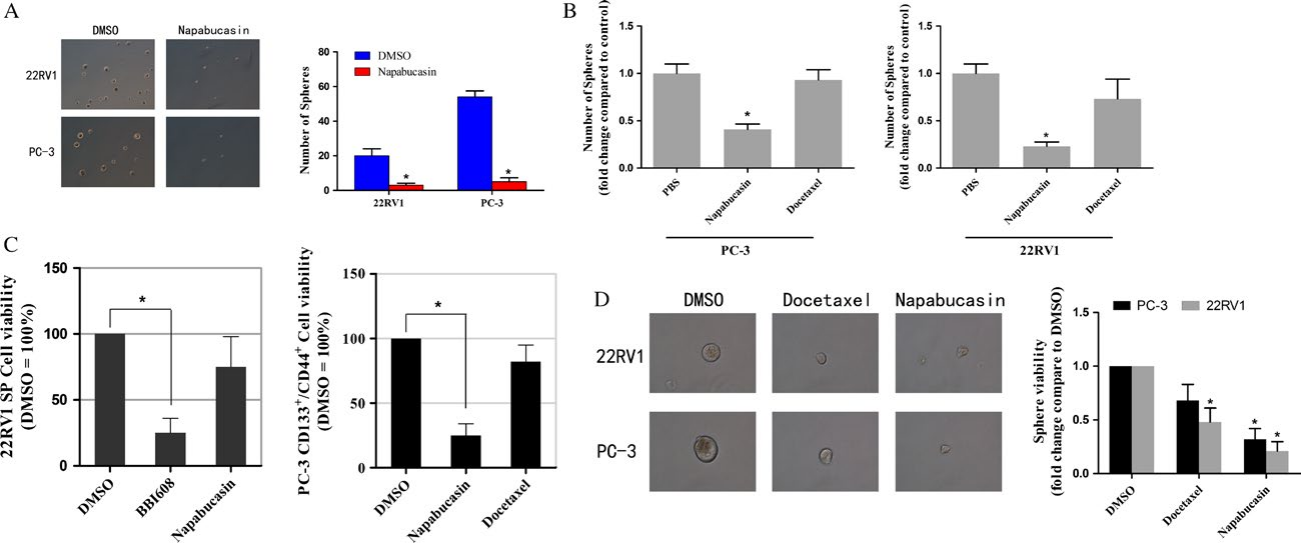
Napabucasin inhibited the self‐renewal of stemness‐high prostate cancer (PCa) cells. The self‐renewal capacity was measured by their ability to grow as spheres and the results showed that the number of spheres was significantly decreased after PCa cells treated with napabucasin in vitro (A) and in vivo (B) (*P *< 0.05; *n* = 3). (C) 22RV1 stemness‐high cells or PC‐3 stemness‐high cells were isolated by FACS based on Hoechst dye exclusion or CD133^+^/CD44^+^ cell surface markers, and were cultured for 72 h in cancer stem cell conditions before the addition of 500 nmol/L napabucasin or 50 nmol/L docetaxel. The cell viability analysis showed that napabucasin depleted the stemness‐high side population (SP) and CD133^+^/CD44^+^ cells (*P *< 0.05; *n* = 3) whereas the side population or CD133^+^/CD44^+^ cells treated with docetaxel had limited effect on viability (*P *> 0.05; *n* = 3). (D) PCa cells were cultured in suspension conditions for 72 h before the addition of 1 *μ*mol/L napabucasin or 100 nmol/L docetaxel, and the quantitative analysis showed that these cells treated with napabucasin resulted in suppression of spherogenesis both in PC‐3 or 22RV1 (*P *< 0.05; *n* = 3) whereas treatment with docetaxel just had obvious effect on the spherogenesis of 22RV1 but not PC‐3 (*P *< 0.05; *n* = 3).

In order to determine the effect of napabucasin on stemness‐high cancer cells in PCa cells, we performed experiments in vitro and in vivo. For in vitro model, cells were firstly treated with 1 *μ*mol/L napabucasin for 24 h and then transferred to ultra low attachment plates in suspension culture medium. The frequency of stemness‐high cancer cells was measured by their ability to grow spheres. Compared with DMSO‐treated cells, napabucasin treatment decreased the stemness‐high cancer cells in PC‐3 and 22RV1 cells by 10‐fold and sevenfold, respectively (Fig. 3A, *P* < 0.05).

For in vivo model, animals were treated with PBS (control), docetaxel, or napabucasin. Tumors were collected aseptically after killing of animals and single‐cell suspensions were disassociated from the collected tumors. As shown in Figure 3B, treatment with napabucasin resulted in inhibition of spherogenesis with numbers of spheres significantly decreased compared with PBS group (*P *< 0.05). However, docetaxel seemed to have no obvious effect on the spherogenesis (Fig. 3B, *P* > 0.05).

### Napabucasin inhibited self‐renewal of PrCSCs isolated by sorting for side population or suspension culture enrichment

To further validate the stemness inhibiting effect of napabucasin, we first isolated or enriched PrCSCs by SP, stem cell surface markers (CD133 and CD44), and suspension culture system, respectively, and then treated with napabucasin. As shown in Figure 3C and D, both the SP cells from 22RV1 cells and CD133^+^/CD44^+^ cells from PC‐3 cells as well as spheres from PC‐3 and 22RV1 cells were obviously inhibited by napabucasin (*P *< 0.05). By contrast, treatment of docetaxel only had obvious effect on the spherogenesis of 22RV1 (*P *< 0.05) but no effect on viability of other PrCSCs (*P *> 0.05).

Moreover, we compared the IC_50_ of napabucasin and docetaxel on PrCSCs and parental bulk cancer cells. As shown in Table 2, PrCSCs from 22RV1 and PC‐3 cells displayed 20‐fold and 180‐fold resistance to docetaxel, respectively. However, the IC_50_ for napabucasin was lower in the PrCSCs than in the bulk cancer cells.

**Table 2:** Comparison of Napabucasin with Docetaxel in normal prostate cancer (PCa) cells and cancer stem cells

PCa cell lines	Compound	IC_50_ (*μ*mol/L)	
		Bulk cells	Cancer stem cells
22RV1	Napabucasin	1.57	1.043
	Docetaxel	0.039	0.785
PC‐3	Napabucasin	3.67	1.35
	Docetaxel	0.017	3.05

### Napabucasin downregulated the expressions of stemness markers

**Figure 4:**
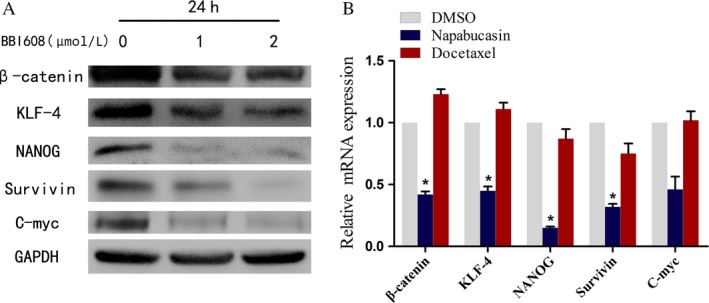
Napabucasin inhibited stemness gene expression. (A) Western Blot and (B) qRT‐PCR analyses showed that the prostate cancer stem cells (PrCSCs)‐related stemness markers involved in the growth and maintenance of PrCSCs was significantly downreguated at protein and mRNA level after treated with napabucasin (*P *< 0.05, *n* = 3).

To assess the effect of napabucasin on stem cell status, we investigated the expressions of several signaling involved in PCa stem cell stemness. As shown in Figure 4A, napabucasin treatment decreased the expression of Nanog, Klf4, survivin, C‐myc, and *β*‐catenin of PrCSCs isolated from PC‐3 cells in a dose‐dependent manner (*P *< 0.05). Furthermore, the qRT‐PCR results also showed a significantly decreased mRNA expression of Nanog, Klf4, survivin, and *β*‐catenin in napabucasin treatment with PC‐3 stemness‐high cancer cells (Fig. 4B, *P* < 0.05).

## Discussion

PCa is the most common cancer and second leading cause of cancer death in men worldwide. Although 5‐year survival rates of PCa have been reported high even in advanced stage, the prognosis of PCa with metastatic disease remains poor because many PCas eventually progress to castration‐resistant prostate cancer (CRPC) with the development of recurrence and metastasis 10.

Current conventional treatments for PCa eliminate most cells within a tumor, but advanced cancers still progress to incurable and metastatic disease 11. Nowadays many studies have proposed that PrCSCs may be a major cause of therapy resistance (including androgen therapy, chemotherapy, and radiotherapy) in advanced PCa because this small population of cells possess unlimited self‐renewal capacities and can regenerate tumorigenic progenies, playing an essential role in PCa therapy resistance, metastasis, and disease relapse 2, 12, 13. Therefore, targeting for PrCSCs may be a promising and effective strategy to combat with CRPC.

However, researches on developing novel therapies for PrCSCs have been hampered by the lack of definitive stem cell markers. Recently, Li et al 8. have identified BBI608 (napabucasin), a potent and promise small‐molecule STAT3 inhibitor (US patent 8,877,803), for the first time and found napabucasin could successfully suppress cancer metastasis and relapse in a variety of solid tumors by inhibition of spherogenesis and killing stemness‐high cancer cells. More importantly, BBI608 seemed to have no adverse effect on hematopoietic or other normal adult stem cells. However, relevant literature about napabucasin was just only one and the effect of napabucasin on PrCSC or PCa cells remains unknown.

To determine the role of napabucasin in PCa, we first examined the effect of napabucasin on PCa cell lines PC‐3 and 22RV1. Our results indicated that napabucasin could inhibit cell proliferation, cell motility, cell survival, colony formation ability, and induced cell apoptosis in vitro and tumorigenesis in vivo. Furthermore, we found that napabucasin could also effectively block sphere formation and kill PrCSCs isolated from PCa in vitro and in vivo. Additionally, the protein and mRNA expression of stem cell markers in stemness‐high PCa cells were decreased after coping with napabucasin. To our knowledge, these are the first data to show the effect of napabucasin on PCa cells and PrCSCs.

At present, docetaxel‐based chemotherapy is widely administered for patients with metastatic (mCRPC) worldwide 14. Although docetaxel confers a significant survival benefit for many patients, all patients inevitably develop resistance to docetaxel and their disease will continue to progress over time. The treatment outcome may be improved by modulating the sensitivity of PCa cells to docetaxel. In this study, our data showed that napabucasin could significantly increase the sensitivity of PCa cells to docetaxel by killing PrCSCs that were drug resistant to docetaxel, suggesting the combination of napabucasin and docetaxel may have complementary and additive antitumor effects on CRPC.

In summary, our data demonstrate that napabucasin significantly inhibits PCa progression and tumorigenesis in vitro and in vivo via suppression of PrCSCs. Future studies should focus on exploring the potential mechanisms of napabucasin, for it could be vital in clinical use. Our findings suggest napabucasin might be a novel and effective way to control both primary and metastatic PCa, specifically CRPC.

## Conflicts of Interest

No potential conflicts of interest were disclosed.

## Supporting information


**Table S1.** The genetic typing results of STR locus and Amelogenin locus of **22RV1**.
**Figure S1.** The STR typing graph of **22RV1**.
**Figure S2.** The intercomparison results of **22RV1** in ATCC.
**Figure S3.** The intercomparison results of **22RV1** in JCRB.
**Figure S4.** The intercomparison results of **22RV1** in DSMZ.

**Table S2.** The genetic typing results of STR locus and Amelogenin locus of **PC‐3**.
**Figure S5.** The STR typing graph of **PC‐3**.
**Figure S6.** The intercomparison results of **PC‐3** in ATCC.
**Figure S7.** The intercomparison results of **PC‐3** in JCRB.
**Figure S8.** The intercomparison results of **PC‐3** in DSMZ.
