# Supplementary material for: Suppression of prostate cancer progression by cancer cell stemness inhibitor napabucasin
Source: Cancer Med. 2016 Feb 21;5(6):1251–8. doi: 10.1002/cam4.675 (PMC4924383; doi:10.1002/cam4.675)

Table 1. The genetic typing results of STR locus and Amelogenin locus of **22RV1**

|  | Allele1 | Allele2 | Allele3 |
| --- | --- | --- | --- |
| D3S1358 | 15 |  |  |
| TH01 | 6 | 9.3 |  |
| D21S11 | 30 |  |  |
| D18S51 | 13 | 14 |  |
| Penta_E | 5 | 13 |  |
| D5S818 | 11 | 12 | 13 |
| D13S317 | 9 | 12 |  |
| D7S820 | 9 | 10 | 11 |
| D16S539 | 12 |  |  |
| CSF1PO | 10 | 11 |  |
| Penta_D | 9 | 12 |  |
| AMEL | x | Y |  |
| vWA | 15 | 21 |  |
| D8S1179 | 12 | 13 | 14 |
| TPOX | 8 |  |  |
| FGA | 20 | 23 |  |

Figure 1 The STR typing graph of **22RV1**


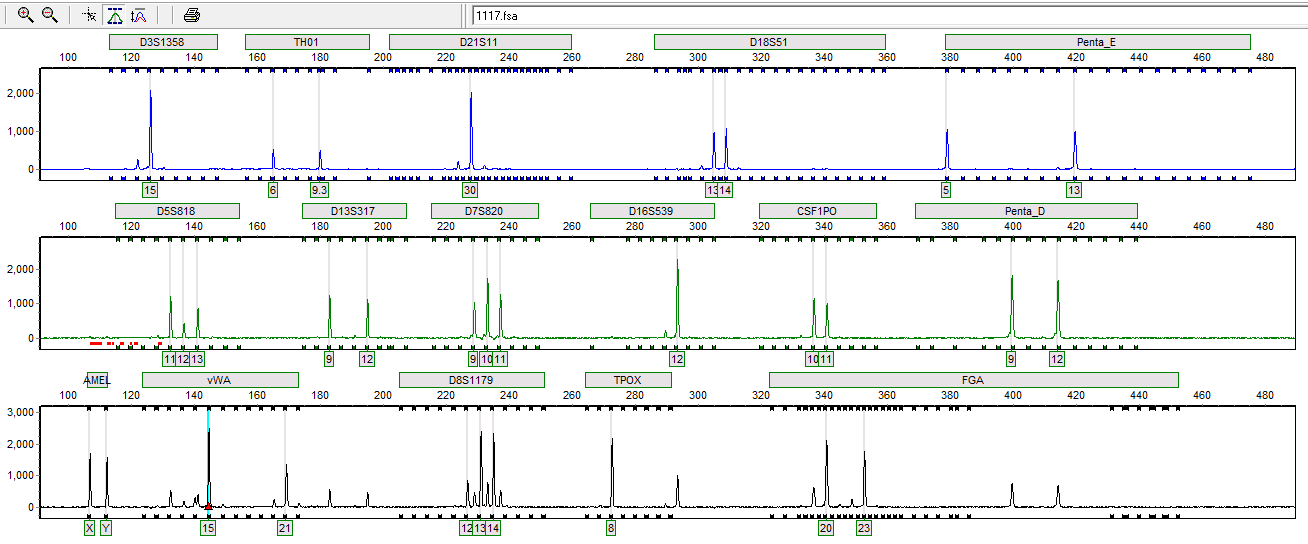


Figure 2 The intercomparison results of **22RV1** in ATCC


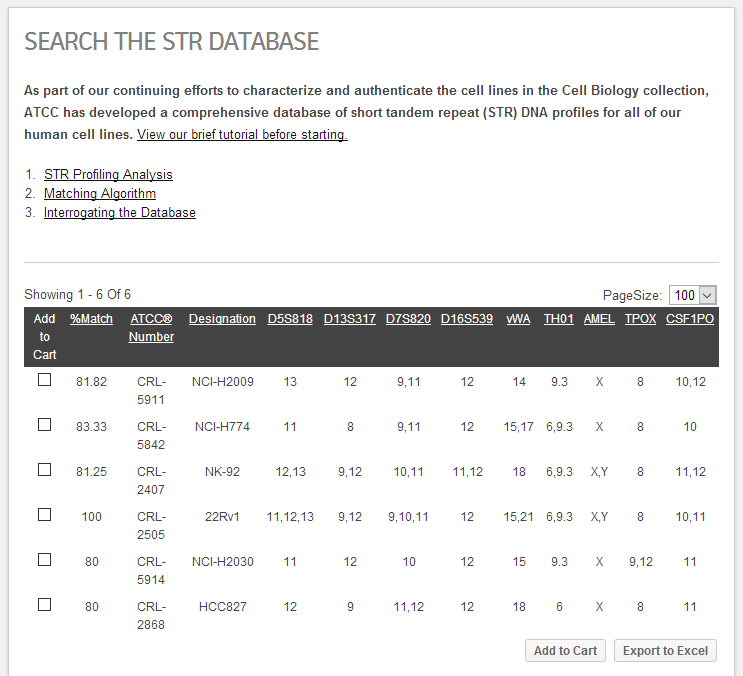


Figure 3 The intercomparison results of **22RV1** in JCRB


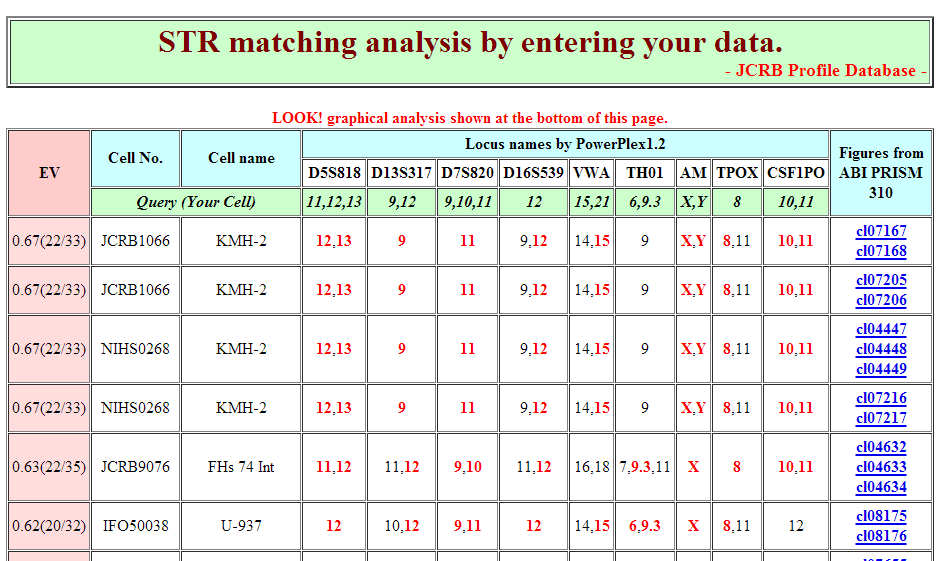


Figure 4 The intercomparison results of **22RV1** in DSMZ


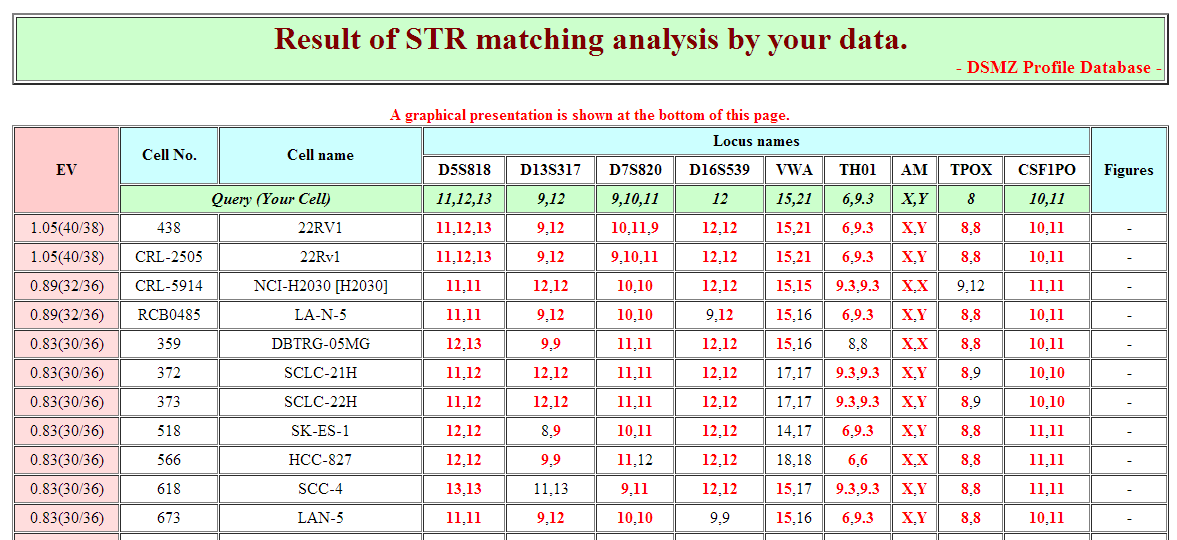

Supplement: Supplementary file 1 — Table S1. The genetic typing results of STR locus and Amelogenin locus of 22RV1. Figure S1. The STR typing graph of 22RV1. Figure S2. The intercomparison results of 22RV1 in ATCC. Figure S3. The intercomparison results of 22RV1 in JCRB. Figure S4. The intercomparison results of 22RV1 in DSMZ. [file CAM4-5-1251-s001.docx]
