# Supplementary material for: Suppression of prostate cancer progression by cancer cell stemness inhibitor napabucasin
Source: Cancer Med. 2016 Feb 21;5(6):1251–8. doi: 10.1002/cam4.675 (PMC4924383; doi:10.1002/cam4.675)

Table 2. The genetic typing results of STR locus and Amelogenin locus of **PC-3**

|  | Allele1 | Allele2 |
| --- | --- | --- |
| D3S1358 | 16 |  |
| TH01 | 6 | 7 |
| D21S11 | 29 | 31.2 |
| D18S51 | 14 | 15 |
| Penta_E | 10 | 17 |
| D5S818 | 13 |  |
| D13S317 | 11 |  |
| D7S820 | 8 | 11 |
| D16S539 | 11 |  |
| CSF1PO | 11 |  |
| Penta_D | 9 |  |
| AMEL | x |  |
| vWA | 17 |  |
| D8S1179 | 13 |  |
| TPOX | 8 | 9 |
| FGA | 24 |  |

Figure 5 The STR typing graph of **PC-3**


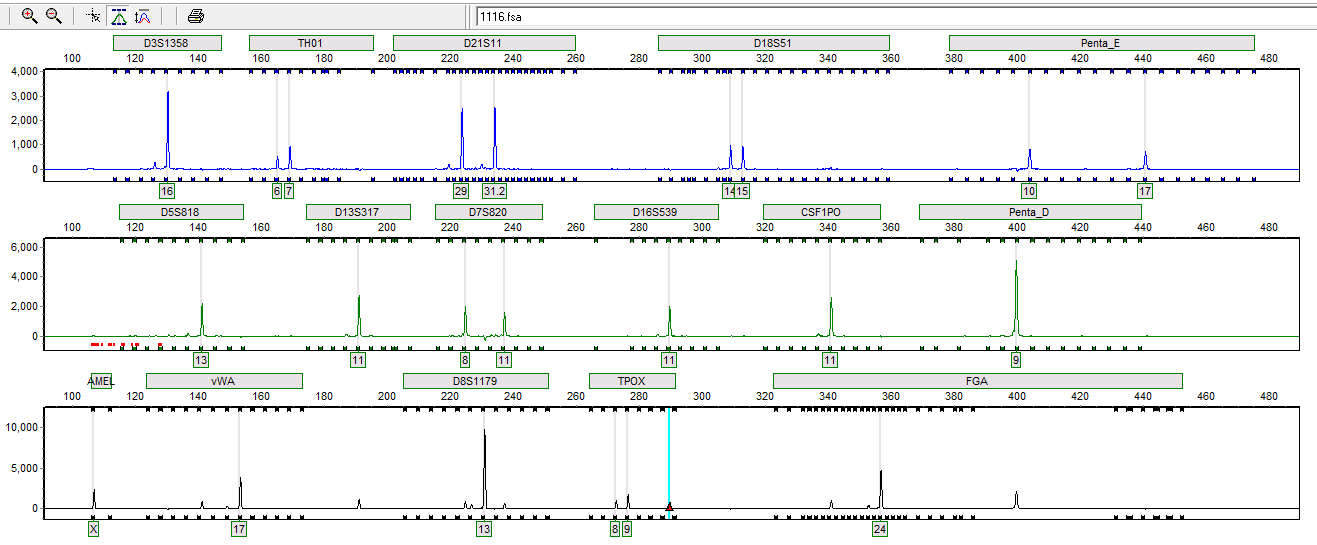


Figure 6 The intercomparison results of **PC-3** in ATCC


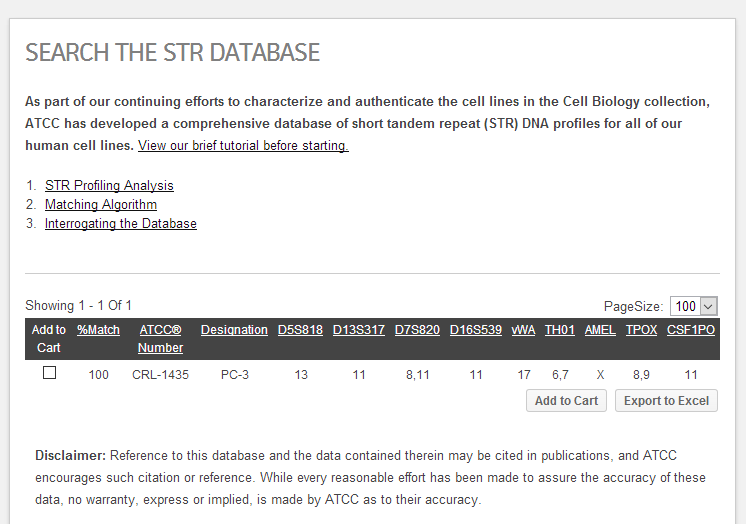


Figure 7 The intercomparison results of **PC-3** in JCRB


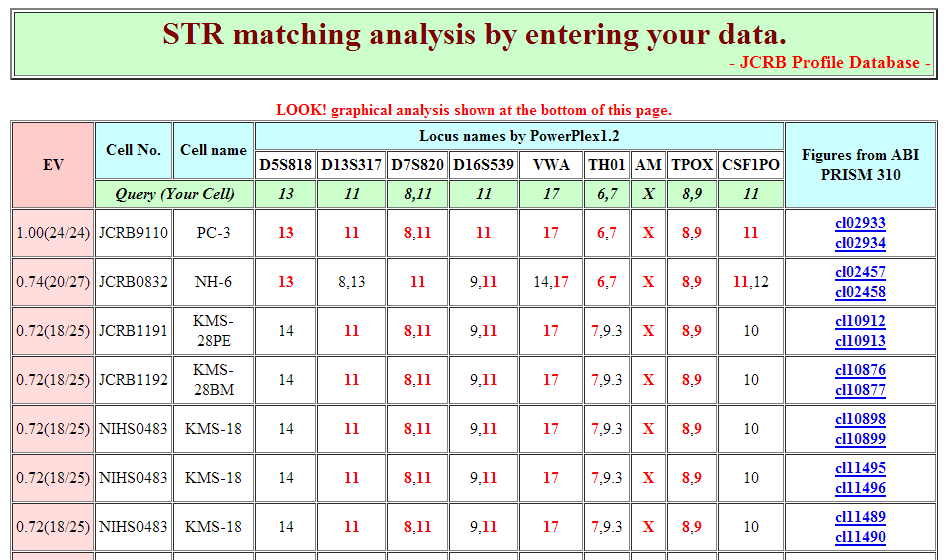


Figure 8 The intercomparison results of **PC-3** in DSMZ


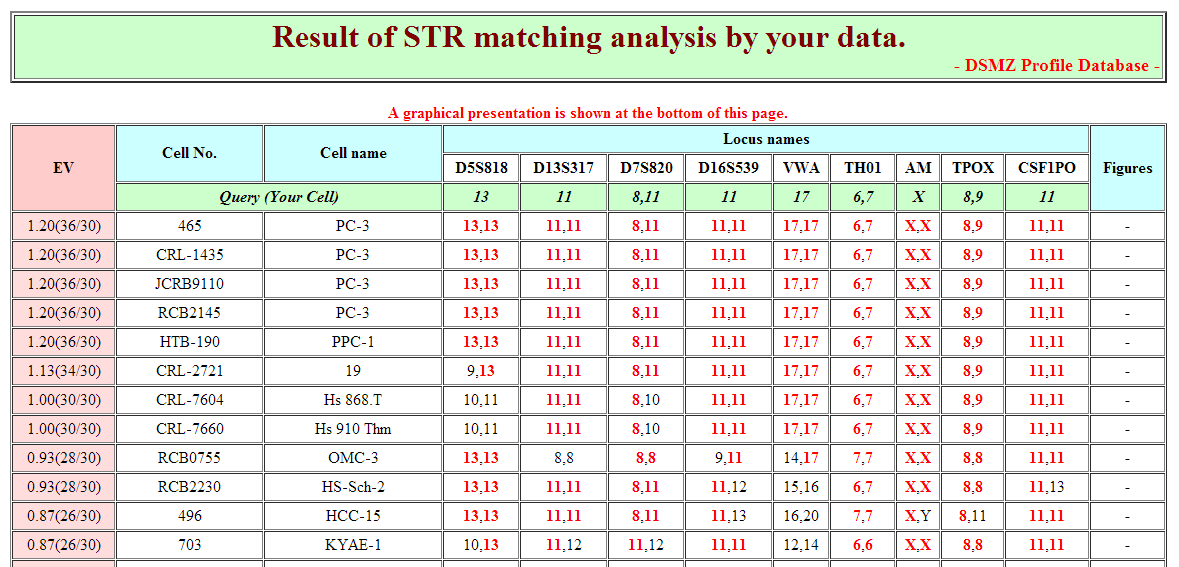

Supplement: Supplementary file 2 — Table S2. The genetic typing results of STR locus and Amelogenin locus of PC‐3. Figure S5. The STR typing graph of PC‐3. Figure S6. The intercomparison results of PC‐3 in ATCC. Figure S7. The intercomparison results of PC‐3 in JCRB. Figure S8. The intercomparison results of PC‐3 in DSMZ. [file CAM4-5-1251-s002.docx]
